# Mid-winter temperatures, not spring temperatures, predict breeding phenology in the European starling *Sturnus vulgaris*

**DOI:** 10.1098/rsos.140301

**Published:** 2015-01-14

**Authors:** Tony D. Williams, Sophie Bourgeon, Allison Cornell, Laramie Ferguson, Melinda Fowler, Raime B. Fronstin, Oliver P. Love

**Affiliations:** 1 Department of Biological Sciences, Simon Fraser University, Burnaby, British Columbia, Canada V5A 1S6; 2 Department of Biological Sciences, University of Windsor, 401 Sunset Avenue, Windsor, Ontario, Canada N9B 3P4

**Keywords:** temperature, breeding phenology, egg-laying date, clutch size, tipulids

## Abstract

In many species, empirical data suggest that temperatures less than 1 month before breeding strongly influence laying date, consistent with predictions that short lag times between cue and response are more reliable, decreasing the chance of mismatch with prey. Here we show in European starlings (*Sturnus vulgaris*) that mid-winter temperature *ca* 50–90 days before laying (8 January–22 February) strongly (*r*^2^ = 0.89) predicts annual variation in laying date. Mid-winter temperature also correlated highly with relative clutch size: birds laid later, but laid larger clutches, in years when mid-winter temperatures were lower. Despite a high degree of breeding synchrony (mean laying date 5–13 April = ±4 days; 80% of nests laid within 4.8 days within year), European starlings show strong date-dependent variation in clutch size and productivity, but this appears to be mediated by a different temporal mechanism for integration of supplemental cue (temperature) information. We suggest the relationship between mid-winter temperature and breeding phenology might be indirect with both components correlating with a third factor: temperature-dependent development of the starling's insect (tipulid) prey. Mid-winter temperatures might set the trajectory of growth and final biomass of tipulid larvae, with this temperature cue providing starlings with information on breeding season prey availability (though exactly *how* remains unknown).

## 2. Introduction

Understanding the relationship between environmental cues and breeding phenology, and the physiological mechanisms mediating these relationships, is essential for predicting organismal responses to climate change [1,2]. In seasonally breeding birds, the timing and duration of breeding are ultimately determined by the food available to support offspring growth during the chick-rearing phase of reproduction [3]. However, seasonal gonadal development and egg laying can be temporally separated from the chick-rearing phase by several weeks or even months owing to the time required for the extensive physiological and behavioural preparation for breeding [4]. Therefore birds, and many other animals, use environmental cues for timing of breeding that can reliably predict, in advance, when food will be plentiful for breeding much later in the season. We have had a working model for control of seasonal reproduction for 40 or more years (reviewed in Williams [4]) which suggests that day length provides reliable ‘initial predictive information’ for general timing of seasonal breeding, perhaps determining a photoperiodic window during which breeding can occur. Then various ‘supplemental factors’ closer to onset of breeding, such as temperature [2,5], fine-tune the actual timing of egg laying to annual variation in *local* breeding conditions (e.g. colder, later, versus warmer, earlier spring temperatures [6–9]).

Most studies to date have shown that temperatures closest to onset of egg laying (less than 1 month), commonly referred to as ‘spring temperatures’, are most highly correlated with laying date [10–12]. This is consistent with our current model of timing of breeding (*sensu* [8,9]) and suggests that spring temperatures are an important supplemental cue determining breeding phenology (although many aspects of temperature cues remain poorly understood [13]). In general, the greater the time-lag between perception of a cue and the fitness consequences of the response to that cue the less informative cues are likely to be to an organism [14,15]. Lag times are important because they increase the probability of mismatches between the environment and the phenotype of the individual [14], and this therefore has important implications for effects of climate change [16,17]. Most studies to date therefore also support this idea that birds use information from supplemental cues with short lag times in order to decrease potential mismatch between the environment and reproductive decisions [14,15,18].

Here we investigated the relationship between pre-breeding temperature and laying date in a species with highly synchronous breeding within- and among years: the European starling (*Sturnus vulgaris*; mean laying date ±4 days among years for first nests). Initially, we predicted (i) that there would be only a weak, or no, relationship between temperature and laying date if photic cues (day length) were sufficient to time synchronous breeding periods, but that (ii) if pre-breeding temperature and laying date were correlated then temperatures closest to onset of egg laying (less than 1 month) would be most highly correlated with laying date, as these would provide the most accurate cue information indicating conditions during egg laying and chick rearing. Although we find a strong, negative correlation between pre-breeding temperature and timing of onset of egg laying, mid-winter temperature (January–February) *ca* 50–90 days before onset of egg laying, not later spring temperature (less than 1 month before), predicted timing of egg laying. These data suggest a complex relationship between temperature, phenology of prey availability and timing of breeding which might explain species-specific variation in responses to climate change (e.g. [19]).

## 3. Material and methods

### 3.1 Breeding data

We used 13 years of breeding data (2002–2014) from our long-term European starling study at Davistead Farm, Langley, British Columbia, Canada (49^°^10^′^ N, 122^°^50^′^ W), which comprises *ca* 150 nest-boxes mounted on posts around pastures and on farm buildings. Each year, we followed the same basic field protocol: nest-boxes were checked daily from 1 April to determine laying date and clutch size, and all newly laid eggs were weighed ( ±0.001 g) and numbered. In several years (2004, 2005, 2007, 2009), we conducted experiments which involved catching females at clutch completion and/or removing eggs to stimulate laying of replacement clutches (e.g. Love and Williams [20]). Therefore, we restricted analysis of laying date and temperature to all ‘first’ clutches initiated during a first peak of egg laying in each year, but restricted analysis of productivity to non-experimental or control birds. We defined this first peak of laying as the 12-day period from the earliest first nest initiation date in any year based on a mean five-egg clutch, two further days for determination of clutch completion and a minimum re-nesting interval after egg removal of 5 days), so that we excluded any potential replacement clutches in experimental years where first clutches were removed (see ‘Discussion’). Experimental birds were banded so we could confirm re-nesting; however, there was also natural re-nesting after early natural breeding failure before we marked birds, so our 12-day cut-off was conservative in excluding these birds too. No first clutches within the first 12 days of laying were excluded from laying date analysis because in experimental years clutches were removed only after completion of laying; therefore, this had no effect on the distribution of laying dates or laying synchrony in years with and without egg removal experiments (and the year of highest synchrony with 80% of nests in 2 days (2012) was a year with no clutch removal).

### 3.2 Temperature data and statistical analysis

Daily temperature data were obtained for the Pitt Meadows weather station, British Columbia (49^°^12^′^ N, 122^°^41^′^ W, elevation 5.0 m.a.s.l.) using the Environment Canada online National Climate Data and Information Archive (http://www.climate.weatheroffice.gc.ca). Pitt Meadows is less than 20 km from our Davistead Farm study site. Mean monthly temperature at Pitt Meadows was highly correlated (*p* ≥ 0.95) with mean monthly and daily temperature at the Cloverdale weather station (20 km south of our study site), and at Vancouver Airport (40 km west), and thus provides a good index of variation in regional temperature.

We used a sliding window approach to determine the time period that provided the best correlation between the average daily temperature and the annual mean laying date [10]. We calculated Pearson's correlations between the mean annual laying dates and the mean, minimum or maximum daily temperature determined using a sliding window, where the window size varied from a minimum 10 days to 80 days for all possible windows between 1 January–31 March (prior to the earliest first-egg date of 1 April; using temperature data for different time periods, e.g. 1 January–30 April, or 1 January–31 December did not change the results). The time period during which the mean temperature provided the highest correlation with the mean laying date was taken to represent the best description of local environmental conditions important for timing of breeding [10]. For example, a window of 1–10 would represent temperatures for 10 days between 1 and 10 January (furthest from onset of egg laying), whereas a window of 81–90 would represent temperatures for 10 days from 22 to 31 March (just before onset of egg laying). We also confirmed the results of the sliding window analysis using a proportional hazard model which describes the probability per time unit that an event (in this case laying date) occurs, as a function of a basic probability (the baseline hazard) and one or more explanatory variables (in this case temperature) and outputs the best fit model [21]. Finally, we conducted univariate analysis of laying date and temperature for particular time periods to test specific hypotheses about how temperature might act as a cue: (i) using mean temperature for August of the preceding year to test for a delayed effect of temperature on larval tipulid survival based on Pearce-Higgins *et al.* [22] (see ‘Discussion’); and (ii) using mean temperature for the period 0–7 days, and 8–15 days prior to the first-egg date in each year, to test for short-term, direct effects of temperature on female reproductive development (see ‘Discussion’). Analyses were carried out using R v. 3.0.1 [23] or SAS v. 9.2 [24].

## 4. Results

Mean laying date for all first nests was 10 April ±3.3 days (*n* = 994 nests, 13 years; table 1), and the earliest and latest mean laying dates among years were 5 April (2010) and 13 April (2008, 2009), respectively, i.e. a range of only 8 days. However, mean egg-laying date varied significantly among years (*F*_12,993_ = 112.2, *p* < 0.0001). As a measure of within-year breeding synchrony, the number of days over which 80% of nests were initiated was 4.8 ± 1.4 days (range 2–8 days).

**Table 1.** Annual variation in first egg date, mean egg-laying date, laying synchrony, clutch size and the slope of the relationship between clutch size and laying date in European starlings, 2002–2014. Values for laying date and clutch size are means ± s.d.

year	nests (*n*)	first egg 1 = 1 Jan	mean first-egg laying date	80% initiation (days)	clutch size	clutch size×date slope (*b*)
2002	72	97	101.5 ± 2.2	5	5.22 ± 0.91	−0.154^**^
2003	106	93	97.5 ± 3.2	8	5.05 ± 0.83	−0.129^***^
2004	97	95	99.1 ± 2.0	5	5.23 ± 0.91	−0.160^***^
2005	97	97	99.4 ± 2.2	5	5.14 ± 0.82	−0.200^***^
2006	58	97	99.4 ± 1.2	3	5.38 ± 0.77	−0.385^***^
2007	71	98	101.7 ± 2.0	5	5.21 ± 1.01	−0.259^***^
2008	67	101	103.3 ± 2.0	5	5.39 ± 0.72	−0.122^**^
2009	72	100	103.3 ± 2.3	5	5.25 ± 0.78	−0.069
2010	69	92	95.0 ± 2.2	5	5.04 ± 0.78	−0.099*
2011	75	98	101.3 ± 2.0	4	5.17 ± 0.74	−0.082
2012	65	99	101.3 ± 1.1	2	5.18 ± 0.75	−0.139
2013	79	92	95.9 ± 2.2	6	5.05 ± 0.89	−0.054
2014	66	100	102.3 ± 2.2	5	5.27 ± 0.85	−0.124^**^

*p < 0.05, ^**^p < 0.01, ^***^p < 0.001.

**Figure 1.**
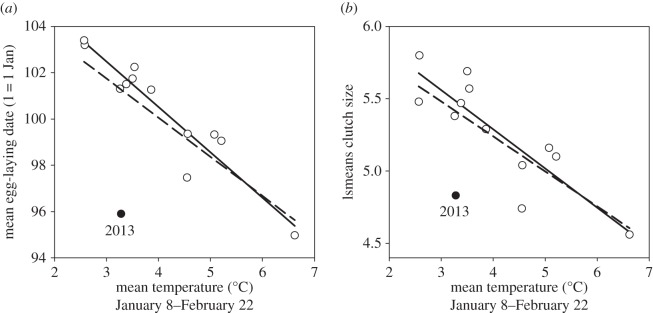
Relationship between mean temperature (^°^C) for the sliding window period 8 January–22 February and (*a*) mean egg-laying date and (*b*) least-squares means (lsmeans) clutch size (corrected for laying date) for 2002–2013. Regression lines are fitted to data with (solid line) and without 2013 (dashed line).

Using data for all years, the sliding window analysis identified a 25-day period from 25 January—19 February which provided the highest correlation between mean ambient temperature and annual mean laying dates (*r*^2^ = 0.53, *p* < 0.01; figure 1*a*, dashed line). However, one year, 2013, was clearly anomalous (see figure 1 and below) and excluding this year the sliding window analysis identified a 45-day period from 8 January to 22 February which provided the highest correlation between mean ambient temperature and annual mean laying dates (*r*^2^ = 0.89, *p* < 0.001): birds laid later in years when mid-winter temperatures 50–90 days before onset of laying were lower (figure 1*a*, solid line). Results were qualitatively very similar using minimum daily temperature (window = 11 January–22 February, *r*^2^ = 0.803, *p* < 0.001) and maximum daily temperature (window = 6 January–21 February, *r*^2^ = 0.933, *p* < 0.001). The proportional hazard model, which used individual breeding data, confirmed that laying dates were statistically significantly influenced by temperature over the period Julian day 8–52 (8 January–21 February; *z* = 19.5, *p* < 0.001).

Mean January temperature alone (1–31 January) was also a good predictor of mean laying date (*r*^2^ = 0.634, *p* < 0.01), but no other mean monthly temperature (February, March, April, August or the preceding year, all *p* > 0.15) predicted annual variation in egg-laying date. Similarly, mean temperature one week (*p* = 0.7) or two weeks (*p* = 0.3) before onset of egg laying in each year was not correlated with mean egg-laying date. Annual variation in mean monthly temperature for March (the pre-breeding period), April (the period of egg laying for first broods) and May (the period of chick-rearing for first broods) was independent of mean temperature for the sliding window period of 8 January–22 February (*p* > 0.25), i.e. the sliding window period was not predictive of temperatures during specific phases of breeding.

Mean clutch size for all years was 5.19 ± 0.84 (range 3–8, *n* = 994; table 1). Clutch size declined by 0.14 ± 0.01 eggs day^−1^ in relation to residual laying date (*F*_1,993_ = 139.9, *p* < 0.001; pooling data for all years), and there was a negative relationship between clutch size and laying date in 9 of 13 individual years (table 1). Clutch size varied significantly among years (*F*_12,993_ = 2.48, *p* < 0.01) controlling for laying date, varying from 4.56 ± 0.23 (least-squares means, in 2010) to 5.80 ± 0.19 (in 2008). However, in marked contrast to the pattern of within-year variation in the clutch size×laying date relationship, least-squares means clutch size was *positively* correlated with mean laying date among-years (*r*_13_ = 0.724, *p* < 0.01). Consequently, adjusted mean clutch size (controlling for laying date) was negatively correlated with mean temperature for the 45-day sliding window period from 8 January–22 February (*r*_13_ = −0.744, *p* < 0.01 including all years; *r*_12_ = −0.867, *p* < 0.001 excluding 2013), i.e. birds laid later, but laid larger clutches, in years when temperatures 50–90 days before egg laying were lower (figure 1*a*,*b*).

## 5. Discussion

European starlings are highly synchronous breeders: at our study site over 13 years mean egg-laying date of first nests only varied by ±4 days among years, and 80% of nests were initiated within a 5-day period within years. This contrasts markedly to the relative asynchronous laying documented for some other species, e.g. initiation of egg laying can occur over a period from several weeks up to 40–50 days [25–28], and in the well-studied great tit (*Parus major*) mean laying date varies by up to one month between years [29] and the range of first egg dates within years averages 27 days (*n* = 59 years; M Visser 2014, personal communication). Within the context of the relative importance of day length versus supplemental factors in determining laying date (*sensu* [8,9]) we therefore expected to find only a weak, or no, relationship between temperature and breeding phenology in the European starling. European starlings are considered one of the more highly photoperiodic species, e.g. showing strong relationships between photoperiod and testis growth [30], and absolute versus relative refractoriness [8,31]. It therefore seemed plausible that this species might rely primarily on the annual cycle of day length to time its synchronous breeding, with little or no modulation by supplemental cues. In fact, we found a very strong (*r*^2^ = 0.89) correlation between pre-breeding temperature and timing of egg laying. However, in contrast to most other species studied to date, laying date was highly correlated with temperatures in January–early February, *ca* 50–90 days prior to onset of laying, not ‘spring’ temperatures less than 1 month before laying [10–12]. Although previous studies of the European starling have concluded that ‘spring’ temperatures influence timing of breeding [32–34], few previous studies have involved formal analysis of long-term datasets to empirically test this idea.

We recognize that some aspects of our analysis could potentially limit interpretation of our data but we do not believe these affect our main conclusions. Firstly, we analysed population means in our sliding window analysis and although the proportional hazard model used individual data we did not consider individuals caught in multiple years, or look at within-individual plasticity of the phenology×temperature response. While this would be an important next step, we have far fewer data on recaptured birds (these represent less than 30% of all individuals recorded each year) and we would argue that the clear-cut results of the population-level analysis (common to other published studies) identifies a phenology temperature pattern very unlike that previously reported. Secondly, we restricted data for first nests to a 12-day period from the earliest first nest initiation date; this was necessary to exclude potential replacement clutches since clearly an analysis of timing of breeding should only include true first clutches. However, this did not affect the distribution of laying dates nor did it generate the high degree of breeding synchrony we document. High breeding synchrony has been reported in other populations of European starlings, e.g. in the UK, although first-egg date varied annually from 9 to 19 April over a 7-year period [33], most first clutches were started within a 10-day period in any one year, and in Belgium all first clutches were laid within 6.7 days (range 4–10 in different years [33]). In our population after this initial 12-day period of first nests, potential new/replacement nests were only initiated at a rate of 0.52 nests day^−1^ (range 0–6) in the first 5-day period after the first peak of laying, and 0.45 nests day^−1^ (range 0–9) in days 6–10 after peak laying. In three years (2012–2014) when we did not remove first clutches, of a total of 274 nests, 78% (*n* = 213) were initiated in the 12-day ‘first nest’ period, and 61 (22%) in the ‘intermediate’ laying period before true second clutches are initiated. However, 31 of the latter nests were known ‘replacement’ clutches, following nest failure, and most of the remaining 30 intermediate nests were initiated in boxes adjacent to boxes with known breeding failure, so were also likely to be replacement clutches. This confirms that natural, late first nests, after the initial 12-day period or egg laying, are very rare in our population.

The range of dates where temperatures are most highly correlated with egg-laying dates varies somewhat among studies and depends in part on species-specific timing of breeding. However, in general, temperatures much closer to onset of egg laying (less than 1 month) most closely predict laying dates [10,11,35] (but see [36]), cf. the *ca* 50–90 days prior to onset of laying in our study, which is therefore up to three times further removed from timing of laying than in most other species. For example, Brommer *et al*. [10] identified a temperature window of 31 March–26 April in the common gull (*Larus canus*) which is less than 1 month from the earliest laying dates of 1 May in this population. Great tits breed relatively earlier and the identified temperature windows in some studies do extend into March, e.g. 1 March–25 April, thus technically including ‘winter’ prior to 21 March [29,37,38]. However, mean laying dates in this species range from 10 April to 10 May so that the temperature window actually includes the mean egg-laying date in about one-third of the years analysed (see [4]), again supporting the idea that temperatures close to onset of laying are more influential. van Balen [38] explicitly considered different time intervals within a broad temperature window of 1 March–21 April in great tits and showed that temperatures for the period 1–20 April, closest to laying dates of 15 April–4 May (less than 1 month), were almost as good a predictor of laying dates (*r* = 0.718) as the period from 1 March, suggesting that earlier, March temperatures are relatively unimportant. In contrast to our results, Meijer *et al*. [39] heated and cooled nest-boxes of captive-breeding European starlings by 2–3^°^C from late March onwards and found that temperature had a direct effect on the timing of laying, suggesting that temperatures just prior to laying were highly important. However, birds were not exposed to elevated temperatures at earlier dates and were provided with food *ad libitum* which might have influenced results. Furthermore, recent studies have highlighted problems and inconsistencies in experimental studies of effects of temperature on timing of laying in captive birds ([40] cf. [41] and [5]), although reconciling differences between laboratories and field studies remains an important challenge for the future [4,13].

**Figure 2.**
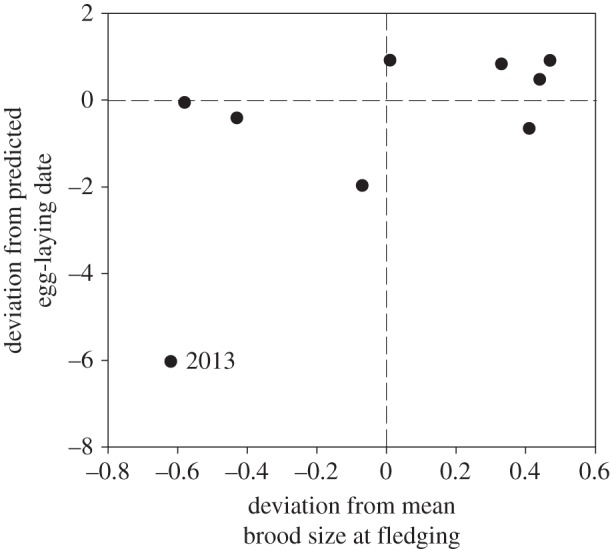
Relationship between deviation in laying date from the predicted laying date (based on the sliding window analysis; laying date = 108.35+(−1.959× temp_window_)) and deviation from overall mean productivity (brood size at fledging).

Despite a very high level of breeding synchrony, clutch size declined with laying date in European starlings in most years of our study (as has been previously documented [33,42]). Smith [42] showed that recruitment success of fledged young also declines very sharply with date, over just a 10-day period in starlings. However, in contrast to previous studies of European starlings, and in contrast to most other species [43], clutch size was positively correlated with annual variation in laying date in our study. Consequently, mid-winter temperatures also predicted relative fecundity, controlling for annual variation in laying date, in our study. Lack [44], citing Kluijver's starling data from Holland (1922–1936), suggested there was no relationship between clutch size and laying date, although the correlation is positive (albeit non-significant) with clutch size increasing from 4.8 to 5.4 eggs over a 14-day range of laying dates, partly supporting our results. A likely explanation for this positive relationship is that in years when laying is initiated earlier females are more likely to produce a second brood, so they lay a smaller ‘sub-optimal’ first clutch to optimize reproductive investment over their multiple breeding attempts [37,45]. Therefore, despite a high level of breeding synchrony, European starlings appear to be exquisitely tuned to ‘date’ as a key factor influencing breeding decisions which, in turn, suggests that starlings should use environmental cues to provide date information. Although we only had one example, in 13 years of data, it seems clear that 2013 was an anomalous year. Mean laying date in 2013 was 6 days earlier than the predicted date based on mid-winter temperatures compared with a maximum deviation in all other years of ±2 days (figure 2). Moreover, brood size at fledging in 2013 was the lowest in our 13-year dataset: compared with a mean for all years (2.51 ± 1.86, *n* = 75 versus 2.96 ± 2.04 chicks, *n* = 350; figure 2). It is tempting to speculate that in 2013 females misinterpreted environmental cues, or the temperature–laying date relationship was uncoupled for other reasons, leading to mismatching with prey [16,17] and hence low productivity.

The mechanism(s) by which temperature affects timing of breeding in birds remains an unresolved question [13]. However, our study found no support for several putative mechanisms. Firstly, egg-laying dates were independent of temperatures one or two weeks before onset of laying, which does not support the hypothesis that temperature has direct effects either on rate of gonadal development (e.g. [46,47]) or on energy expenditure of resource acquisition of pre-laying females [48]. Tinbergen [49] also found no correlation between temperature in the 10 or 20 days preceding laying and onset of laying in starlings in Holland over 7 years. Thus, temperature is unlikely to function to ‘lift a constraint’ on earlier reproduction [40] in starlings. Secondly, the main prey item of European starlings during breeding is tipulid (cranefly) larvae [49,50], including at our study site *Tipula paludosa*. In another tipulid-dependent species, the European golden plover (*Pluvialis apricaria*), breeding productivity is correlated with cranefly abundance which, in turn, is correlated with August temperature in the *previous* year [22]. Pearce-Higgins *et al*. [22] suggested that the mechanism for temperature effects on golden plovers is likely to be through changes in cranefly populations, with high summer temperature causing rapid population declines because of the vulnerability of newly deposited eggs and early larval instars to desiccation. Although this example specifically considered breeding productivity of plovers, our discussion below suggests that temperature can (somehow) affect both timing of tipulid larval development and final biomass, so this could potentially affect timing and productivity in starlings. Nevertheless, we found no evidence to support this hypothesis in starlings, at least for timing of breeding: August temperatures of the preceding year were not correlated with subsequent laying date.

So, how can mid-winter temperature be linked to breeding decisions (date of egg laying) in females almost 3 months later? In some avian species, the timing of migration is correlated with spring temperatures along the migration route, before birds reach the breeding grounds (e.g. [51]), and this may act as a mechanism to match annual differences in phenology with the timing of breeding. However, this mechanism does not apply in our study: our population of European starlings is resident or only partially, but locally, migratory. We catch birds banded during breeding at our study site throughout November to March (approx. 20% of sampled birds are re-traps, similar to our return rate across breeding seasons), and our only banding recoveries outside the breeding season have been within 100 km of the study site (Washington State, USA). So individual birds would experience local winter temperatures and could use these as a proximate cue. Meijer *et al.* [39] reported that a warmth sum calculated for the first four weeks in March was negatively correlated (*r* = 0.8) with annual variation in laying date in European starlings over a 10-year period in southern Germany, but this population of European starlings is migratory, arriving at the colony in the second half of February, and Meijer *et al*. [39] therefore did not include earlier temperatures in their analysis. However, this highlights the obvious prediction that local temperature cues during the non-breeding period can only be important in resident species. Nevertheless, in our study population mid-winter temperatures have no value as predictive cues of later spring temperatures since they were not correlated with temperature during April (egg laying) or May (chick-rearing). Instead, we suggest that the relationship between mid-winter temperature and breeding phenology is indirect, with both these components correlating with a third factor: temperature-dependent development of the starling's main prey. European starlings mainly feed their chicks tipulid (cranefly) larvae, or other soil larvae [49,50], which comprise more than 80% of prey items fed to chicks at our study site. Tipulid larvae have a limited period of availability (approx. 30–40 days), especially at peak larval biomass, in late April/May [32] during which many of our birds attempt to produce two broods (see fig. 3.3 in [4]), and this is probably key in explaining the high breeding synchrony. In particular, tipulid growth is temperature-sensitive [32,52,53] with growth rate being slowest at low temperatures in December–February. Both growth rate and size of larvae appear to be determined by the field conditions to which they are exposed *early* in their development, and larval growth rate is positively related to asymptotic larval weight [54]. Timing of development is also temperature-sensitive: mean date of third moult depends on the date soil starts to warm up in spring—which is presumably affected by how cold soil temperatures are in January and February—usually occurring two to three weeks after the temperature reaches 5^°^C [52]. Furthermore, there is some evidence for ‘critical’ temperature periods early in development: *Tipula montana* larvae maintained at 1^°^C during their second instar and then transferred to 7^°^C had *ca* 50% lower final weights than larvae maintained at 5^°^C as second instars [55]. It therefore seems plausible that mid-winter temperatures might set the trajectory of development and final biomass of tipulid larvae, such that this temperature cue provides European starlings with information on breeding season prey availability (though exactly *how* remains unknown).

Our data have important implications for mechanistic models of how supplemental cues should work to fine-tune phenology [13]. In addition, given the long lag time between cue (mid-winter temperature) and response (egg laying in April), our results pose a challenge to our theoretical understanding of how environmental cues are used (cf. [14,15]) and have important implication for how some (synchronous?) species might respond in the future to climate change. Longer lag times, as in the European starling, should lead to weaker correlations in climatic conditions between the environment of decision making and that of selection (*sensu* [1]), making these cues less informative, and increasing the likelihood of mismatching.

## Data accessibility

Data available from the Dryad repository, doi:10.5061/dryad.1jt86.
